# Stability and change in life satisfaction in Japan before and during the COVID‐19 pandemic

**DOI:** 10.1111/aphw.70021

**Published:** 2025-03-27

**Authors:** Takeshi Nakagawa, Taiji Noguchi, Ayane Komatsu, Xueying Jin, Sayaka Okahashi, Tami Saito

**Affiliations:** ^1^ Research Institute National Center for Geriatrics and Gerontology Obu Aichi Japan; ^2^ Graduate School of Human Sciences Osaka University Suita Osaka Japan

**Keywords:** lifespan, longitudinal, novel coronavirus, protective factors, risk factors, well‐being

## Abstract

The outbreak of the novel coronavirus (COVID‐19) significantly impacted individuals' daily lives and may provide meaningful implications for well‐being. This study examined how individuals' well‐being changed before and during the COVID‐19 pandemic and explored the potential risk and protective factors for well‐being. A total of 15,574 Japanese adults aged 15 to 89 years participated in baseline online surveys in February 2019 or February 2020 (*n* = 10,293 in the 2019 sample and *n* = 5,281 in the 2020 sample). Both samples were followed up in 2021 and 2022. Well‐being was indexed as a single‐item indicator of life satisfaction. Piecewise growth models demonstrated that life satisfaction typically remained stable before and during the pandemic. Risk and protective factors for life satisfaction were consistent across samples overall. Individuals perceiving better economic satisfaction and self‐rated health following the pandemic and without a support network before the pandemic showed greater increases in life satisfaction after the outbreak. Our findings suggest that most individuals' well‐being did not deteriorate following the pandemic but that some individuals might have lost or even gained resources for life satisfaction. To better understand resilience and well‐being, researchers should consider how the COVID‐19 pandemic has changed multiple aspects of daily lives.

## INTRODUCTION

The outbreak of the novel coronavirus (COVID‐19) and subsequent government responses to prevent the spread of the infectious disease, including quarantine, stay‐at‐home orders, and social distancing, significantly impacted individuals' daily lives worldwide. Researchers and clinicians voiced the urgent need to address mental health problems (Gruber et al., 2021; Pfefferbaum & North, 2020; Van Bavel et al., 2020). In line with their concerns, empirical studies have revealed the adverse consequences of COVID‐19 on negative components of well‐being, such as depressive symptoms, during the early months of the pandemic (Dragioti et al., 2022; Jin et al., 2021; Prati & Mancini, 2021; Wu et al., 2021). However, most of the initial evidence was based on cross‐sectional studies conducted in North America and Western Europe shortly after the outbreak, which has prevented us from drawing generalizability outside of Western countries and clear causal inferences regarding the impact of the pandemic. Also, earlier studies have considered the multi‐dimensionality of well‐being (e.g., cognitive and affective well‐being; Diener, 1984) and observed differential patterns of changes according to components of well‐being (Aknin, Andretti, et al., 2022): Cognitive aspects of well‐being, such as life satisfaction, remained largely stable throughout the first year of the pandemic, while affective aspects of well‐being deteriorated just after the outbreak.

To better understand well‐being during the pandemic, this study used data collected from 2019 to 2022 to examine how individuals' well‐being changed in Japan before and during the COVID‐19 pandemic while identifying the potential risk and protective factors for well‐being. We indexed well‐being as life satisfaction, which is regarded as a key positive component of well‐being (Diener, 1984) and an indicator for planning and assessing the impact of policy decisions (Organisation for Economic Co‐operation and Development, 2013). As described below, findings on life satisfaction will help researchers and practitioners better understand how individuals adapt to stressful circumstances accompanied by the pandemic and identify groups at high risk of maladaptation. Furthermore, policymakers could evaluate the effectiveness of infection control measures not only from the medical and economic aspects but also from the psychological aspect by accumulating comparative evidence on life satisfaction across countries. Consequently, investigating life satisfaction amid the pandemic would provide valuable insights into theory, practice, and policy.

## TRAJECTORIES OF LIFE SATISFACTION DURING THE COVID‐19 PANDEMIC

Longitudinal data collected before and during the pandemic are important for inferring the effects of the pandemic outbreak. While some studies operationalized data obtained after the outbreak of the COVID‐19 pandemic as mid‐ or post‐pandemic data and reported a deterioration in life satisfaction using pre‐ and mid‐pandemic data (Bezzo et al., 2021; Wanberg et al., 2020), other studies found that life satisfaction remained stable or minimally declined, suggesting negligible effects of COVID‐19 (Hettich et al., 2022; Milicev et al., 2022; Wettstein et al., 2023; Wettstein, Nowossadeck, et al., 2022; Wettstein, Wahl, et al., 2022). However, the majority of evidence has been accumulated in North America and Western Europe, such as the United States, the United Kingdom, and Germany. Considering cross‐country variations in policy responses to COVID‐19 (Brauner et al., 2021; Haug et al., 2020), there is insufficient evidence to draw firm conclusions about whether life satisfaction remained unchanged or declined in the face of the pandemic outbreak. Considering that the stability and recovery of well‐being in adversity are regarded as resilience (Luthar & Cicchetti, 2000), understanding changes in well‐being and potential factors promoting well‐being during the pandemic can offer implications for the promotion of resilience.

## RISK AND PROTECTIVE FACTORS FOR LIFE SATISFACTION DURING THE COVID‐19 PANDEMIC

The abovementioned longitudinal evidence has also revealed substantial individual differences in trajectories of life satisfaction during the COVID‐19 pandemic. Some individuals were more vulnerable and exhibited steeper declines in life satisfaction, whereas others were more resilient and demonstrated quicker recovery. Thus, researchers have explored potential risk and protective factors for life satisfaction to identify vulnerable individuals and allocate public health resources to them. Considering the impact of lockdown and restriction measures imposed during the outbreak on individuals' daily lives, such as changing the routine of the families, causing conflicts among family members, and reducing face‐to‐face social contacts and out‐of‐home activities, most studies have applied a so‐called bottom‐up approach (Diener, 1984; Diener et al., 1999), in which levels of individuals' well‐being are presumed to be influenced by life circumstances, including sociodemographic, social, economic, and health‐related factors. These multiple aspects of factors have been presumed as important causes of well‐being in research before the outbreak of COVID‐19 (Diener, 1984; Diener et al., 1999).

However, the scarcity of longitudinal studies with pre‐ and mid‐pandemic assessment has hindered examining how changes in risk and protective factors in the face of the pandemic are associated with well‐being. Some studies reported that unfavorable health conditions, such as poor self‐rated health and mental illness, before the pandemic were related to deteriorating life satisfaction (Benke et al., 2022; Wettstein, Nowossadeck, et al., 2022). Other studies suggested that changes in social and economic factors covaried with life satisfaction during the pandemic (Kalseth et al., 2023; Milicev et al., 2022; Wels et al., 2022), but the associations of social and economic factors with life satisfaction are somewhat mixed across studies. Counterintuitively, a few studies (Milicev et al., 2022; Wanberg et al., 2020) indicated that better social and economic factors could harm life satisfaction during the pandemic.

According to the conservation of resources theory (Hobfoll, 1989, 2002), some individuals are likely to experience a loss of resources for well‐being under overriding stressful circumstances. Consequently, resilient individuals who possess resources before such circumstances may become vulnerable due to the loss of resources. To better understand the role of changing life circumstances on well‐being during the pandemic, the conservation of resources theory (Habfoll, 1989, 2002) offers implications that changes in risk and protective factors following the pandemic should be incorporated. Drawing on the bottom‐up approach (Diener, 1984; Diener et al., 1999) and the conservation of resources theory (Habfoll, 1989, 2002), we thus examined the associations of a variety of resources—sociodemographic, social, economic, and health‐related—available before the COVID‐19 pandemic and their changes thereafter with trajectories of well‐being accompanied by the pandemic. We predicted that a loss of resources would be related to decreases in well‐being.

## THE CURRENT STUDY

Despite cross‐country variations in infection control measures, longitudinal studies with pre‐ and mid‐pandemic assessments remain limited except for North America and Western Europe. Thus, we do not know yet whether existing evidence about life satisfaction before and during the COVID‐19 pandemic is generalizable across countries with different cultural values as well as public health policies (Zhang et al., 2022). Furthermore, only a scarcity of studies have investigated the role of changing life circumstances on life satisfaction. This study aimed to extend prior research by examining how life satisfaction changed in East Asia, namely Japan, before and during the COVID‐19 pandemic (i.e., from 2019 to 2022).

In Japan, infection and mortality rates were relatively low during the pandemic compared with other countries (Onozuka et al., 2022). This lower burden of COVID‐19 could be partly due to cultural values. People in collectivistic countries, such as East Asia, were more likely to use masks compared with those in individualistic countries, such as North America (Lu et al., 2021). In contrast, the patterns of regional disparities in Japan, where the COVID‐19 outcomes were severe in regions with low socioeconomic status, were similar to those in North America and Western Europe (Yoshikawa & Kawachi, 2021). Nevertheless, it remains unclear if the pandemic disproportionately impacted the well‐being of individuals who resided in regions with high COVID‐19 prevalence and whether and to what extent such regional differences are observed across countries. Some studies in the United Kingdom and Germany indicated that geographical differences in life satisfaction were negligible (Milicev et al., 2022; Wettstein, Nowossadeck, et al., 2022), while one in South Korea found decreases in life satisfaction in a region where the virus spread more extensively (Kim et al., 2023). Considering such cross‐national similarities and differences in COVID‐19 outcomes, the evidence on life satisfaction in Japan would provide new insights for evaluating the effectiveness of infection control measures from psychological and sociocultural perspectives.

We further explored whether risk and protective factors available before the pandemic and their changes from pre‐ to mid‐pandemic periods were associated with trajectories of life satisfaction. Given that the conservation of resources theory (Hobfoll, 1989, 2002) presumes that some individuals can lose resources under stressful circumstances, we predicted that a loss of resources, in particular social and economic, following the outbreak would be related to declines in life satisfaction. Our findings from Japan may yield unique insights considering low infection and death rates from COVID‐19 despite moderate social distancing measures and the high ratio of older adults (Iwasaki & Grubaugh, 2020).

## METHODS

### Participants and procedure

We did not apply for ethics approval for the current study because we used publicly available deidentifying datasets. Participants were drawn from the Survey on Satisfaction and Quality of Life conducted by the Cabinet Office of Japan (2022). The study design is illustrated in Figure S1. To evaluate the robustness of our findings, we separately analyzed two longitudinal datasets with three measurement observations of one sample who participated in the 2019, 2021, and 2022 surveys, and another sample who participated in the 2020, 2021, and 2022 surveys (as described below, the 2019 sample and the 2020 sample, respectively).

The participants were recruited through online survey panels and were offered a monetary incentive for their participation. Recruitment was stratified by age, sex, and geographical region. The survey initially adopted an annually repeated cross‐sectional design. A total of 10,293 participants responded to the first survey administered from January to February 2019. In February 2020, just after the first case of COVID‐19 was reported on January 16, 5,281 participants responded to the second survey. In March 2021 and February 2022, the participants of the first and second surveys were followed up. A total of 4,540 and 6,105 participants responded to the third and fourth surveys, respectively. Figure S2 shows the trends in daily new confirmed COVID‐19 cases in Japan from 2020 to 2022. Whereas the third survey in 2021 was conducted under the government's emergency declaration stated only in one Metropolitan region (i.e., Tokyo and its neighboring prefectures) during the declining phase of the pandemic, the fourth survey in 2022 was conducted under the semi‐state of emergency in more than 70% of prefectures during the peak phase of the pandemic.

Table 1 summarizes the descriptive characteristics of the 2019 and 2020 samples at baseline and follow‐up surveys. The number of participants in each measurement observation is shown in Figure S1. The 2019 sample provided an average of 1.54 observations per person (*SD* = 0.69), and the 2020 sample responded with a mean of 1.96 observations per person (*SD* = 0.87).

**TABLE 1:** Descriptive characteristics of two study samples.

		2019 sample (*n* = 10,293)		2020 sample (*n* = 5,281)			Test of the difference	
Variables		*M* or *n*	*SD* or %	*M* or *n*	*SD* or %	Range	Statistics	*p*
Age in 2019 or 2020	15 to 24	1,792	17.4%	879	16.6%		χ^2^(5) = 8.11,	.150
	25 to 34	1,837	17.8%	912	17.3%		Cramer's *V* = .023	
	35 to 44	1,959	19.0%	992	18.8%			
	45 to 54	1,563	15.2%	778	14.7%			
	55 to 64	1,755	17.1%	986	18.7%			
	65+	1,387	13.5%	734	13.9%			
Sex	Male	5,102	49.6%	2,611	49.4%		χ^2^(1) = 0.22, *φ* = .001	.881
	Female	5,191	50.4%	2,670	50.6%			
Education	Low	3,388	32.9%	1,656	31.4%		χ^2^(2) = 5.30,	.071
	Medium	2,392	23.2%	1,298	24.6%		Cramer's *V* = .018	
	High	4,513	43.8%	2,327	44.1%			
Region in 2019 or 2020	Metropolitan region	3,287	31.9%	1,820	34.5%		χ^2^(1) = 10.13, *φ* = .026	.001
	Non‐metropolitan region	7,006	68.1%	3,461	65.5%			
Residential status In 2019 or 2020	Living alone	1,643	16.0%	878	16.6%		χ^2^(1) = 1.13, *φ* = .009	.287
	Living with others	8,650	84.0%	4,403	83.4%			
Support network in 2019 or 2020	No	990	9.6%	488	9.2%		χ^2^(1) = 0.58, *φ* = .006	.447
	Yes	9,303	90.4%	4,793	90.8%			
Paid job in 2019 or 2020	No	2,655	25.8%	1,126	21.3%		χ^2^(1) = 37.98, *φ* = .049	<.001
	Yes	7,638	74.2%	4,155	78.7%			
Economic satisfaction	2019 or 2020	4.67	2.34	4.75	2.33	0–10	*t*(15,572) = 2.20, Cohen's *d* = .037	.028
	2021	4.83	2.38	4.94	2.27		*t*(4,538) = 1.46, Cohen's *d* = .045	.144
	2022	4.98	2.36	4.99	2.31		*t*(6,103) = 0.12, Cohen's *d* = .003	.904
Self‐rated health	2019 or 2020	2.33	1.03	2.34	1.04	0–4	*t*(15,572) = 2.31, Cohen's *d* = .004	.817
	2021	2.25	1.02	2.27	1.00		*t*(4,538) = 0.58, Cohen's *d* = .018	.565
	2022	2.15	1.02	2.20	1.00		*t*(6,103) = 1.79, Cohen's *d* = .048	.074
Life satisfaction	2019 or 2020	5.78	2.34	5.83	2.31	0–10	*t*(15,572) = 1.14, Cohen's *d* = .019	.256
	2021	5.60	2.35	5.77	2.29		*t*(4,538) = 2.37, Cohen's *d* = .073	.018
	2022	5.74	2.39	5.78	2.32		*t*(6,103) = 0.62, Cohen's *d* = .016	.537

*Note*: Two study samples participated in online baseline surveys in 2019 or 2020 and were followed up in 2021 and 2022. Higher values indicate better levels of economic satisfaction, self‐rated health, and life satisfaction. The metropolitan region includes three major cities in Japan: Tokyo, Osaka, and Nagoya.

To verify the patterns of missing values due to dropout during the follow‐up period, we compared the participants who responded to the baseline survey only and those who continued to participate in the follow‐up survey. Descriptive characteristics of the continuers and dropouts in the two samples are presented in Tables S1 and S2. Across the 2019 and 2020 samples, education and residential status did not statistically differ between the participants who dropped out and those who continued. However, the continuers were more likely to be older, male, have no support network, have no paid job, and reported poorer health status than the dropouts, with minor differences (*φ*s ≤ |0.05|or Cohen's *d* ≤ |0.10|), except for age (*φ* = 0.22 for the 2019 sample and *φ* = 0.28 for the 2020 sample). Regarding life satisfaction, differential patterns were observed between the two samples: Whereas life satisfaction was slightly lower in the continuers than in the dropouts in the 2019 sample (Cohen's *d* = —0.05), it did not differ between the continuers and dropouts in the 2020 sample (Cohen's *d* = 0.00).

### Measures

#### Life satisfaction

Using a single‐item measure of global life satisfaction, we assessed life satisfaction as the outcome variable. At each survey, participants were asked, “In general, how satisfied are you with your present life?” with an 11‐point Likert scale ranging from 0 (*not at all satisfied*) to 10 (*completely satisfied*). Empirical studies demonstrated the reliability and validity of single‐item measures of life satisfaction (Cheung & Lucas, 2014; Lucas & Donnellan, 2012). In addition, the single‐item measure has been widely used across countries (Huppert et al., 2009), which enhances the comparability of results across studies.

In the subsequent analyses, we recorded life satisfaction scores into a *T* metric (*M* = 50 and *SD* = 10) using the baseline data of the 2019 sample as the reference frame (*M* = 5.78, *SD* = 2.34). Based on Cohen's (1992) guidelines for interpreting effect sizes, a difference of 2 *T*‐score points represents a small effect, and a difference of five points represents a medium effect.

#### Risk and protective factors

Based on the bottom‐up approach (Diener, 1984; Diener et al., 1999) and the previous evidence reviewed above, we included the following sociodemographic, social, economic, and health‐related factors for life satisfaction: age, sex, education, region of residence, residential status, support network, paid job, economic satisfaction, and self‐rated health. As described in the “Data Analysis” section, categorical variables were effect‐coded for contrast with the grand means of all groups instead of dummy‐coded for contrast with a reference group. Similarly, continuous variables were centered at the sample means in the subsequent analyses.

Regarding time‐invariant baseline variables, age was not measured in years but assessed as a categorical variable with six age groups: 15 to 24 years, 25 to 34 years, 35 to 44 years, 45 to 54 years, 55 to 64 years, and 65 years and older. Specifically, to compare the mean of the focal group and the grand mean of all groups, we effect‐coded age as −1 for the reference group (e.g., *15 to 24 years*), 1 for the focal group (e.g., *25 to 34 years*), and 0 for all other groups. We created five effect‐coded variables for age. Sex was coded as −1 (*male*) and 1 (*female*). Education was categorized into low, middle, and high, based on the International Standard Classification of Education (ISCED) 2011 (UNESCO, 2012): Low education represented ISCED levels two and three, or lower to upper secondary education; middle education represented ISCED levels four and five, or post‐secondary to short‐cycle tertiary education; and high education represented ISCED levels 6 or more, or Bachelor's or above level. Similar to age groups, to compare the mean of the focal group and the grand mean of all groups, we effect‐coded education as −1 for the reference group (e.g., *low education*), 1 for the focal group (e.g., *middle education*), and 0 for the remaining group (e.g., *high education*). Two effect‐coded variables were created for education. Participants' prefectures of residence were classified into metropolitan and non‐metropolitan regions. The regions of residence were coded as −1 (*metropolitan region*) and 1 (*non‐metropolitan region*). The metropolitan region included the three largest areas of Tokyo and its neighboring prefectures (i.e., Ibaraki, Saitama, Chiba, Kanagawa), Aichi and its neighboring prefecture (i.e., Mie), and Osaka and its neighboring prefectures (i.e., Kyoto, Hyogo, and Nara). The number of household members was assessed, and residential status was coded as −1 (*living alone*) and 1 (*living with others*). To assess the support network, participants were asked the question, “Do you have family or friends you could ask for help when you needed it?” Support network was coded as −1 (*no*) and 1 (*yes*). Work status (e.g., a part‐time worker, a full‐time worker, a self‐employed worker, and a student) was assessed, and having a paid job was coded as −1 (*no*) and 1 (*yes*).

In terms of time‐varying variables, participants responded to a single‐item measure of economic satisfaction with an 11‐point Likert scale ranging from 0 (*not at all satisfied*) to 10 (*completely satisfied*) at each survey. To assess self‐rated health, participants were asked, “How do you feel about your current health status?” and responded to a 5‐point scale from 0 (*poor*) to 4 (*good*). We considered changes in economic satisfaction and self‐rated health in the pre‐ and mid‐pandemic periods, as explained below in the “Data Preparation” section.

### Data preparation

To consider changes in the time‐varying variables in the pre‐ and mid‐pandemic periods, we used the piecewise growth model (Ram & Grimm, 2007; Singer & Willett, 2003) and extracted information about pre‐pandemic levels and mid‐pandemic changes in economic satisfaction and self‐rated health. The model was specified as follows:

(1) $$\text{time}-{\text{varying variables}}_{\mathrm{ti}}=\beta_{0i}+\beta_{1i}\left(\mathrm{mid}-{\text{pandemic}}_{\mathrm{ti}}\right)+e_{\mathrm{ti}},$$

In Equation 1, person *i*'s time‐varying variables at time *t* are modeled as the function of an individual‐specific intercept parameter that indicates levels before the COVID‐19 pandemic, $\beta_{0i}$; an individual‐specific parameter representing differences in levels before and during the pandemic, $\beta_{1i}$; and a residual error, $e_{\mathrm{ti}}$.

Furthermore, Individual‐specific parameters were modeled as follows:

(2) $$\begin{array}{c} \beta_{0i}=\gamma_{00}+\mu_{0i},\\ \beta_{1i}=\gamma_{10}+\mu_{1i}, \end{array}$$

In Equation 2, $\gamma_{00}$ and $\gamma_{10}$ indicate sample means (fixed effects), and $\mu_{0i}\;$and $\mu_{1i}$ represent individual deviations from the means (random effects). The individual differences, $\mu_{0i}\;$and $\mu_{1i}$, are presumed to follow a multivariate normal distribution, to correlate with each other, and to be uncorrelated with the residual errors, $e_{\mathrm{ti}}$.

Table S3 shows the results of the piecewise growth model for time‐varying variables (i.e., economic satisfaction and self‐rated health) in the 2019 and 2020 samples. The patterns of the changes were consistent across samples: whereas self‐rated health, on average, worsened following the COVID‐19 pandemic, economic satisfaction improved from the pre‐ to mid‐pandemic periods. Based on these models, we extracted the random effect parameters of pre‐ and mid‐pandemic economic satisfaction and self‐rated health and further standardized the mid‐pandemic parameters so that one unit corresponds to 1 *SD*. Then, as described below in the “Data analysis” section, we included the two parameters (i.e., levels before the pandemic and differences from the pre‐ to mid‐pandemic periods) of each time‐varying variable as predictors of trajectories of life satisfaction accompanied by the COVID‐19 pandemic.

### Data analysis

To examine the trajectories of life satisfaction before and during the COVID‐19 pandemic, we estimated a linear growth model and a piecewise growth model. Figure S3 graphically illustrates hypothetical trajectories of life satisfaction based on the two models. In the linear model, we assumed that life satisfaction changed linearly over time to/from the pandemic and operationalized the time metric as the years to/from the onset of the COVID‐19 pandemic (i.e., centered in early 2020). Thus, the time metrics were coded as −1, 1, and 2 for the 2019 sample and 0, 1, and 2 for the 2020 sample. In the piecewise model, we presumed that life satisfaction exhibited different levels between the pre‐ and mid‐pandemic periods and aimed to compare average levels before and during the pandemic. To do so, we created a mid‐pandemic time metric representing the period after the outbreak of the pandemic. The time metric was coded as 0 (*pre‐pandemic*) for 2019 and 2020 and 1 (*mid‐pandemic*) for 2021 and 2022 across samples. Thus, in the piecewise growth model, the mid‐pandemic metric indicates differences in the mean levels between the pre‐ and mid‐pandemic periods.

After creating the time metrics, we examined the average trajectories of life satisfaction by fitting unconditional growth models that only included time metrics as predictors. The unconditional linear growth model was specified as follows:

(3) $${\text{life satisfaction}}_{\mathrm{ti}}=\beta_{2i}+\beta_{3i}\left({\text{time in study}}_{\mathrm{ti}}\right)+e_{\mathrm{ti}},$$

In Equation 3, person *i*'s life satisfaction at time *t* is modeled as a function of an individual‐specific intercept parameter that indicates levels in the first survey in 2019, $\beta_{2i}$; an individual‐specific slope parameter representing the linear rate of change since 2019, $\beta_{3i}$; and residual error, $e_{\mathrm{ti}}$.

Individual‐specific parameters were modeled as follows:

(4) $$\begin{array}{c} \beta_{2i}=\gamma_{20}+\mu_{2i},\\ \beta_{3i}=\gamma_{30}+\mu_{3i}, \end{array}$$

Next, the unconditional piecewise growth model was specified as Equation 1 indicated. Thus, person *i*'s life satisfaction at time *t* is modeled as the function of an individual‐specific intercept parameter that indicates levels before the COVID‐19 pandemic, $\beta_{0i}$; an individual‐specific parameter representing differences in levels before and during the pandemic, $\beta_{1i}$; and a residual error, $e_{\mathrm{ti}}$.

Individual‐specific parameters were modeled as Equation 2 indicated. In Equations 2 and 4, $\gamma_{00}$, $\gamma_{10}$, $\gamma_{20}$, and $\gamma_{30}$ represent sample means. $\mu_{0i}$, $\mu_{1i}$, $\mu_{2i}$, and $\mu_{3i}$ are individual deviations from the means. The individual differences, $\mu_{0i}$, $\mu_{1i}$, $\mu_{2i}$, and $\mu_{3i}$ are assumed to follow a multivariate normal distribution, to correlate with each other, and to be uncorrelated with the residual errors, $e_{\mathrm{ti}}$.

After estimating the linear and piecewise growth models in the 2019 and 2020 samples, we compared fit statistics between the models in each sample and selected the better‐fitting model. We then examined risk and protective factors of trajectories of life satisfaction before and during the COVID‐19 pandemic by fitting conditional growth models with added predictors (i.e., time‐varying variables obtained in the previous “Data Preparation” section and time‐invariant baseline variables). In our primary analysis, economic satisfaction and self‐rated health were included as time‐varying variables, and the remaining factors were considered time‐invariant. This is because, while the abovementioned two variables were continuous, other variables were categorical. Therefore, we had to choose data obtained from continuers who participated in the pre‐ and mid‐pandemic surveys for examining the associations of changes in categorical predictors with life satisfaction, which led to substantial reductions in the sample sizes (see the “Follow‐up analysis” for details). Nevertheless, we further conducted follow‐up analyses to examine the time‐varying effects of residential status, support network, and paid job. Across conditional models, predictors were effect‐coded for categorical variables and centered at the sample means for continuous variables so that parameters estimated in the conditional models indicated differences from the grand mean of the samples. Effect coding contrasts different groups, and the coefficients can be interpreted as the differences between a specific group mean and the grand mean of all groups (for coding details, see Wendorf, 2004).

When fitting the models for the outcome and time‐varying variables, we adopted maximum likelihood estimation to handle the missing values due to dropouts using the standard missing‐at‐random assumption (Little & Rubin, 2002). We reported unstandardized estimates and interpreted effect sizes. Statistical significance was defined at *p* < .05 (two‐tailed). We estimated models using the “nlme” package (Pinheiro et al., 2019) in *R* and the MIXED program in IBM SPSS.

## RESULTS

### Trajectories of life satisfaction before and during the COVID‐19 pandemic

To examine changes in life satisfaction in relation to the onset of the COVID‐19 pandemic, Table S3 summarizes the results of the unconditional linear and piecewise growth models in the 2019 and 2020 samples. When comparing model fit indices, fit statistics for the linear and piecewise models were equivalent in the 2019 sample (Akaike information criterion [AIC] = 114,648.58 for the linear model; AIC = 114,648.52 for the piecewise model). In the 2020 sample, the piecewise model fitted better to the data than the linear model (AIC = 73,052.98 for the linear model; AIC = 73,036.76 for the piecewise model). We also compared the two models based on the proportional reduction of explained within‐person variance (i.e., changes in pseudo *R* ^2^; Snijders & Bosker, 1999) when adding either time to/from pandemic or mid‐pandemic time metric to the random intercept‐only models without any predictors. In the 2019 sample, given that the residual within‐person variance of the intercept‐only model was 33.37, the changes in pseudo *R* ^2^ for the linear and piecewise models were equivalent (∆pseudo‐*R* ^2^ = .21 and .23 for the linear and piecewise models, respectively). In the 2020 sample, given that the residual within‐person variance of the intercept‐only model was 29.74, the change in pseudo *R* ^2^ for the piecewise model was slightly larger than that for the linear model (∆pseudo‐*R* ^2^ = .04 and .11 for the linear and piecewise models, respectively). In sum, the fit statistics in the 2020 sample suggest that the piecewise growth model with the mid‐pandemic time metric fitted better to the data and more appropriately described the changes in life satisfaction that took place at the onset of the COVID‐19 pandemic than did the linear growth model with the time to/from pandemic.

The fixed effects In Table S4 refer to the estimated average changes in life satisfaction before and during the pandemic. Across the samples and models, life satisfaction, on average, did not change statistically significantly in relation to the pandemic. The linear models showed that individuals' life satisfaction remained relatively stable over time across the samples (0.01 and −0.05 *T*‐score units per year in the 2019 and 2020 samples, respectively). Similarly, the piecewise models showed that life satisfaction exhibited statistically negligible differences between the pre‐ and mid‐pandemic periods (−0.00 and −0.16 *T*‐score units between the two periods in the 2019 and 2020 samples, respectively). Figure S4 depicts prototypical piecewise changes in life satisfaction following the pandemic outbreak.

The random effects in Table S4 present the interindividual variabilities from the average changes in life satisfaction before and during the COVID‐19 pandemic. The linear models showed minor or negligible interindividual differences in the rate of change in life satisfaction ($\sigma_{\mu_{3}}^{2}$ = 2.14 and 1.17 in the 2019 and 2020 samples, respectively), indicating that approximately 68% of the participants were distributed in a range of ±1.46 and ±1.08 in the 2019 and 2020 samples, respectively, from the average rate of changes. In contrast, the piecewise models presented moderate interindividual variabilities in the degree of differences from pre‐ to mid‐pandemic periods ($\sigma_{\mu_{1}}^{2}$ = 18.40 and 8.48 in the 2019 and 2020 samples, respectively), suggesting that about 68% of the participants were distributed in a range of ±4.29 and ±2.91 in the 2019 and 2020 samples, respectively, from the average differences.

### Risk and protective factors of life satisfaction before and during the COVID‐19 pandemic

**FIGURE 1:**
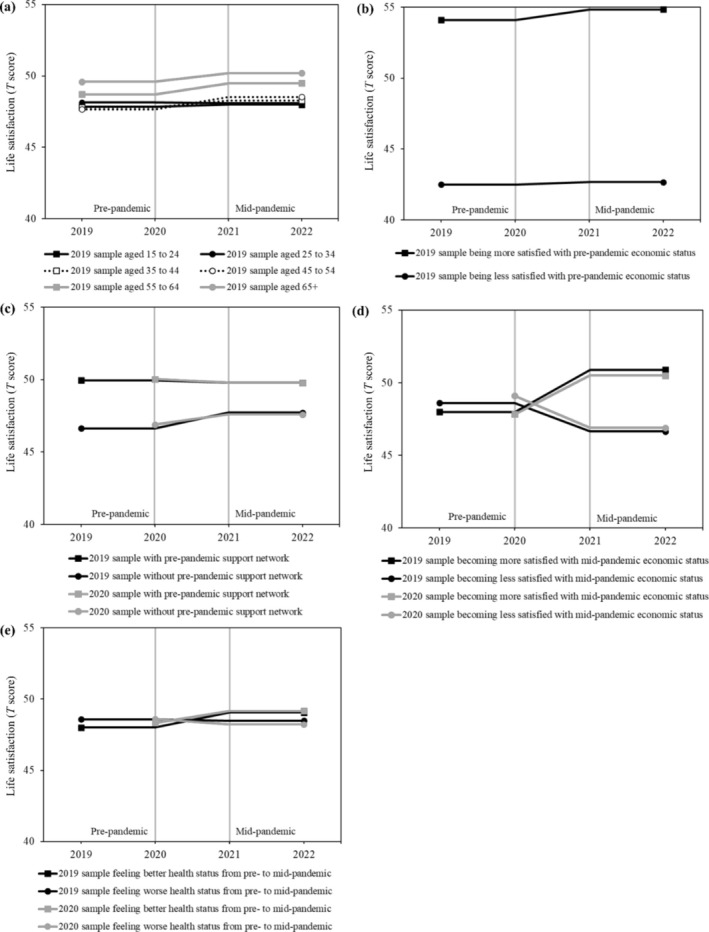
Risk and protective factors for life satisfaction before and during the COVID‐19 pandemic. Differential patterns of piecewise changes in life satisfaction between the pre‐ and mid‐pandemic periods are illustrated in figures based on conditional models with predictors. See coefficients in Table 2 for details. On average, the two samples demonstrated small or negligible increases in life satisfaction following the pandemic outbreak after controlling for predictors. (a) Individuals aged 25 to 34 showed less steep or negligible increases in life satisfaction from the pre‐ to mid‐pandemic periods compared with those of the average age group. (b) Individuals who were more satisfied with their economic status before the pandemic (+1*SD*) demonstrated steeper increases during the mid‐pandemic period than those who were less satisfied (−1*SD*). (c) Individuals without a support network before the pandemic showed steeper increases in life satisfaction from pre‐ to mid‐pandemic compared with those with a support network. (d) Individuals who became more satisfied with their economic status after the pandemic (+1 unit) exhibited greater increases from pre‐ to mid‐pandemic compared with those who became less satisfied (−1 unit). (e) Individuals who reported better health after the pandemic (+1 unit) displayed steeper increases in the mid‐pandemic period than those who reported poorer health (−1 unit).

Table 2 summarizes the results of the conditional piecewise models that examine the potential risk and protective factors of changes in life satisfaction before and during the COVID‐19 pandemic. On average, the study samples displayed small or negligible increases in life satisfaction following the onset of the pandemic after controlling for the predictors (Estimate = 0.47 and 0.23 in the 2019 and 2020 samples, respectively). Based on the parameters estimated in Table 2, Figure 1 illustrates the associations of the factors with the trajectories of life satisfaction in relation to the pandemic. As described below, the relevant factors were overall consistent across the 2019 and 2020 samples, although a few were observed only in the 2019 sample.

**TABLE 2:** Piecewise growth models for life satisfaction: conditional models with predictors.

		2019 sample (*n* = 10,293)		2020 sample (*n* = 5,281)	
		Pre‐pandemic intercept	Mid‐pandemic	Pre‐pandemic intercept	Mid‐pandemic
		Estimate (*SE*)		Estimate (*SE*)	
Fixed effects		48.29 (0.14)***	0.47 (0.19)*	48.46 (0.20)***	0.23 (0.22)
Age in 2019 or 2020	15 to 24	−0.44 (0.16)**	−0.34 (0.26)	−0.74 (0.22)***	−0.22 (0.30)
	25 to 34	−0.16 (0.15)	−0.52 (0.22) *	−0.46 (0.22)*	0.15 (0.11)
	35 to 44	−0.50 (0.15)***	0.01 (0.20)	−0.47 (0.21)*	−0.00 (0.23)
	45 to 54	−0.63 (0.16)***	0.40 (0.22)	0.21 (0.23)	−0.09 (0.25)
	55 to 64	0.41 (0.15)**	0.32 (0.21)	0.33 (0.21)	0.18 (0.22)
	65+	1.31 (0.18)***	0.13 (0.25)	1.14 (0.25)***	−0.01 (0.27)
Sex	Male	−0.50 (0.07)***	0.06 (0.10)	−0.39 (0.10)***	0.04 (0.12)
	Female	0.50 (0.07)***	−0.06 (0.10)	0.39 (0.10)***	−0.04 (0.12)
Education	Low	−0.01 (0.10)	−0.07 (0.10)	−0.16 (0.14)	0.04 (0.16)
	Medium	−0.07 (0.11)	0.11 (0.16)	0.03 (0.16)	0.04 (0.18)
	High	0.08 (0.10)	−0.03 (0.14)	0.13 (0.14)	−0.08 (0.15)
Region in 2019 or 2020	Metropolitan region	0.11 (0.08)	−0.07 (0.11)	−0.02 (0.10)	−0.05 (0.12)
	Non‐metropolitan region	−0.11 (0.08)	0.07 (0.11)	0.02 (0.10)	0.05 (0.12)
Residential status in 2019 or 2020	Living alone	−0.60 (0.10)***	0.11 (0.14)	−0.57 (0.13)***	0.08 (0.15)
	Living with others	0.60 (0.10)***	−0.11 (0.14)	0.57 (0.13)***	−0.08 (0.15)
Support network in 2019 or 2020	No	−1.66 (0.12)***	0.63 (0.16)***	−1.56 (0.17)***	0.48 (0.19)*
	Yes	1.66 (0.12)***	−0.63 (0.16)***	1.56 (0.17)***	−0.48 (0.19)*
Paid job in 2019 or 2020	No	−0.06 (0.09)	0.12 (0.12)	−0.16 (0.13)	0.09 (0.14)
	Yes	0.06 (0.09)	−0.12 (0.12)	0.16 (0.13)	−0.09 (0.14)
Economic satisfaction	Pre‐pandemic	3.29 (0.05)***	0.16 (0.06) *	3.18 (0.06)***	0.10 (0.07)
	Mid‐pandemic	−0.31 (0.08)***	2.43 (0.08) ***	−0.64 (0.10)***	2.44 (0.10)***
Self‐rated health	Pre‐pandemic	2.13 (0.11)***	−0.16 (0.15)	1.99 (1.35)***	0.24 (0.17)
	Mid‐pandemic	−0.29 (0.08)***	0.57 (0.08) ***	−0.14 (0.11)	0.60 (0.11)***
Random effects (variance–covariance matrix)					
	Pre‐pandemic intercept	23.33 (1.20)***		23.27 (1.27)***	
	Mid‐pandemic	−7.04 (1.24)***	6.13 (2.04)**	−2.28 (1.19)	0.66 (1.71)
	Residual variance	25.75 (0.98)***		26.23 (0.83)***	
Goodness of fit					
	−2*LL*	104,959.78		68,074.20	
	*AIC*	105,035.78		68,150.20	

*Note*: Unstandardized estimates (standard errors in parentheses) are shown. Pre‐ and mid‐pandemic predictors were estimated using piecewise growth models. In both the 2019 and 2020 samples, life satisfaction score is standardized to a *T* metric (*M* = 50, *SD* = 10) based on baseline data of the 2019 sample. The intercept is centered in early 2020 when the first case of COVID‐19 was reported in Japan. Categorical predictors were effect‐coded so that each coefficient represents a deviation from the grand mean of the samples. Continuous predictors were centered at the sample means. Mid‐pandemic economic satisfaction and self‐rated health are standardized. Positive values indicate better levels of life satisfaction than the average levels. –2*LL* = −2 log likelihood; *AIC* = Akaike information criterion.

* *p* < .05

** *p* < .01

*** *p* < .00.

Statistically significant differences in age groups and pre‐pandemic economic satisfaction were observed only in the 2019 sample. The increase was less steep and negligible in individuals aged 25 to 34 than in those of the average age group (Estimate = 0.47–0.52 = −0.05; see Figure 1a). Additionally, individuals who were more satisfied with their economic status before the pandemic showed steeper increases in life satisfaction thereafter. Based on Cohen's (1992), however, the differences before and during the COVID‐19 pandemic were small (Estimate = 0.47 + 0.16 = 0.63; see Figure 1b).

Across the 2019 and 2020 samples, we found minor differences between individuals who had a support network prior to the pandemic and those who did not. Individuals without a support network exhibited—counterintuitively—slightly steeper increases in life satisfaction from the pre‐ to mid‐pandemic periods than those with a support network (Estimate = 0.47 + 0.63 = 1.10 in the 2019 sample, and Estimate = 0.23 + 0.48 = 0.71 in the 2020 sample; see Figure 1c).

Changes in perceived economic and health status from the pre‐ to mid‐pandemic periods were related to changes in life satisfaction across samples. Individuals who became more satisfied with their economic status after the pandemic outbreak reported greater increases in life satisfaction (Estimate = 2.43 and 2.44 in the 2019 and 2020 samples, respectively; see Figure 3d). Those who rated their health status better after the outbreak also demonstrated steeper increases in life satisfaction (Estimate = 0.57 and 0.60 in the 2019 and 2020 samples, respectively; see Figure 1e).

Regarding the other predictors, we found non‐significant, negligible interindividual differences in changes in life satisfaction associated with sex, education, region of residence, residential status, and having a paid job across the 2019 and 2020 samples.

### Follow‐up analysis

In our analysis reported above, we included time‐varying continuous variables (i.e., economic status and self‐rated health). In the follow‐up analysis, we further included additional time‐varying categorical variables (i.e., residential status, support network, and paid job), using data obtained from continuers who participated in the pre‐ and mid‐pandemic surveys. Specifically, we created and effect‐coded the time‐varying categorical predictors using data collected in the baseline (i.e., 2019 or 2020) and follow‐up (i.e., 2021) surveys. As presented in Table S5, residential status, support network, and having a paid job from pre‐ to mid‐pandemic periods did not change for most participants.

The results of the follow‐up analysis are summarized in Table S6. The model converged only in the 2019 sample. None of the time‐varying categorical predictors were statistically associated with changes in life satisfaction from pre‐ to mid‐pandemic periods. However, individuals who did not have a support network before the pandemic but gained a new support network during the pandemic were likely to exhibit increases in life satisfaction (Estimate = 1.34 + 0.97 = 2.31; see the results of fixed effects in Table S6), but the differences from the pre‐ to the mid‐pandemic periods were small. Additionally, those who continued to have a support network displayed less steep or negligible increases (Estimate = 1.34–0.39 = 0.95). Changes in perceived economic and health status were robustly related to trajectories of life satisfaction across models, whereas age differences did not demonstrate consistent patterns.

## DISCUSSION

In this study, we examined how life satisfaction changed in Japan before and during the COVID‐19 pandemic, while exploring the role of risk and protective factors available before the pandemic and their changes from the pre‐ to mid‐pandemic periods. Longitudinal data obtained from the two samples yielded overall robust patterns of results. Below, we first discuss trajectories of life satisfaction accompanied by the pandemic.

### Trajectories of life satisfaction before and during the COVID‐19 pandemic

As the unconditional piecewise models indicated (see Figure S4), this study demonstrated that life satisfaction, on average, remained unchanged across samples. These findings are consistent with several previous studies (Hettich et al., 2022; Milicev et al., 2022; Wettstein et al., 2023; Wettstein, Nowossadeck, et al., 2022; Wettstein, Wahl, et al., 2022) that found that most individuals adapted to the circumstances accompanied by the pandemic. Considering the timing of the assessments after the pandemic outbreak (i.e., March 2021 and February 2022) and an earlier study reporting the recovery of life satisfaction by December 2020 (Ishida & Ishida, 2021), individuals might have already adapted to the pandemic within one year following the pandemic outbreak. However, we also observed substantial individual differences in changes in life satisfaction from pre‐ to mid‐pandemic periods. As Table S4 suggested, most individuals maintained their pre‐pandemic levels of life satisfaction during the pandemic, but in some individuals, life satisfaction deteriorated, while for others, it even improved. Additionally, considering that the piecewise model fitted better to the data than the linear model, our results suggest that some individuals faced discontinuities around the onset of the COVID‐19 pandemic and exhibited abrupt changes in their daily lives just thereafter.

Regarding the conditional piecewise models, the results showed that life satisfaction increased slightly or negligibly following the onset of the pandemic. The differences in life satisfaction from the pre‐ to mid‐pandemic periods between the unconditional and conditional models would be due to effect coding for categorical predictors. Specifically, categorical variables (i.e., residential status, support network, and paid job) were time‐varying in nature but were included as time‐invariant predictors in the conditional models. Thus, the average differences from the pre‐ to mid‐pandemic periods estimated in the conditional models represented those of the grand mean of all groups, which were presumed to remain unchanged over time. Therefore, when estimating the prototypical changes before and during the COVID‐19 pandemic, the results of the unconditional models should be interpreted.

Taken together, these findings highlight the importance of practice and policy in identifying vulnerable individuals following the occurrence of adversity and allocating public health resources to them. Furthermore, practitioners and policymakers should endeavor to promote individuals' resilience even under stressful circumstances. Below, we discuss both risk and protective factors of life satisfaction in detail.

### Risk and protective factors of life satisfaction before and during the COVID‐19 pandemic

According to the conservation of resources theory (Hobfoll, 1989, 2002), individuals tend to lose resources that support their well‐being under extremely stressful circumstances. However, only a few studies have directly assessed changes in resources from pre‐ to mid‐pandemic periods and have yielded incongruent evidence regarding the role of resources for life satisfaction: Some studies suggested that the loss of social and economic resources could trigger declines in life satisfaction (Kalseth et al., 2023; Milicev et al., 2022; Wels et al., 2022), supporting the conservation of resources theory. In contrast, other studies (Milicev et al., 2022; Wanberg et al., 2020) indicated that available resources may have harmful effects on life satisfaction during the pandemic. This study yielded both results that supported and did not support the conservation of resources theory and provided a more nuanced picture of how individuals adapted to the pandemic than the theoretical prediction.

First, this study demonstrated that changes in resources before and during the COVID‐19 pandemic were robustly associated with life satisfaction across samples: Declines in economic and health‐related resources have covaried with a deterioration in life satisfaction (see Figure 1d and e). These findings supported the hypothesis based on the conservation of resource theory (Hobfoll, 1989, 2002) and prior research indicating that poor pre‐pandemic levels of economic resources and subsequent shrinkage could result in a deterioration in life satisfaction (Benke et al., 2022; Wels et al., 2022; Wettstein, Nowossadeck, et al., 2022). Furthermore, economic factors were most strongly associated with trajectories of life satisfaction accompanied by the pandemic outbreak among the four relevant factors—sociodemographic, social, economic, and health‐related—. Thus, our results reveal that, whereas most individuals became more satisfied with their economic status following the pandemic, a few individuals facing economic disruptions were particularly vulnerable to COVID‐19‐related impacts. Practitioners and policymakers should consider the trade‐offs between public health and the economy when confronting future pandemics and implementing infection control measures, including lockdown and social distancing policies. In Japan, however, while self‐rated health, on average, worsened following the pandemic, economic satisfaction improved (see Table S3). The COVID‐19 pandemic could result in a loss of health‐related resources even due to the implementation of non‐coercive lockdowns, but financial aid programs might preserve individuals' economic resources. For example, the Japanese government implemented several financial aids, including direct financing and credit guarantee systems, in response to the severe initial impact of the outbreak. Consequently, numerous small and medium‐sized enterprises as well as low‐income households could utilize these public financial aids to compensate for revenue deficits during the pandemic. The causal effects of specific measures in response to COVID‐19 on multiple aspects of daily lives should be systematically investigated.

Next, individuals without a support network before the pandemic reported lower levels of life satisfaction in the pre‐pandemic period than those with a support network; however, counterintuitively, life satisfaction recovered or even improved after the onset of the pandemic. This pattern was robust across samples (see Figure 3c), albeit with small differences. These findings did not support the conservation of resources theory that presumes the protective role of resources in times of stressful circumstances (Hobfoll, 1989, 2002). We do not know the underlying mechanisms, but several potential pathways could exist. First, individuals without social resources might have sought and gained new support during the pandemic, as our follow‐up analysis suggested (see Table S6). Even vulnerable individuals who do not have resources may become resilient and gain new resources under stressful circumstances. Second, individuals with social resources could have been more likely to experience conflict due to social distancing mandates to prevent the transmission of COVID‐19 (Pietromonaco & Overall, 2021; Prime et al., 2020). Our follow‐up analysis indicated that, despite statistically non‐significant results, individuals with a support network continuously might experience less steep increases in life satisfaction compared to those of the grand mean of all groups (Estimate = 1.34–0.39 = 0.95; see Table S6). Thus, the protective role of social resources might have been outweighed during the pandemic. A previous study indicated that life satisfaction of individuals who possessed social resources might decline (Milicev et al., 2022). However, studies have not provided sufficient empirical findings to draw clear conclusions regarding family processes and relationships during the COVID‐19 pandemic.

There were also slightly inconsistent results across samples. Younger adults aged 25 to 34 showed less steep increases in life satisfaction than those of the average age group in the 2019 sample (see Figure 1a), but such age differences were not found in the 2020 sample. Due to the categorical measure of age groups, we might not accurately capture age differences in life satisfaction. Nevertheless, our results were at least consistent with previous findings (Benke et al., 2022; Milicev et al., 2022; Wettstein, Nowossadeck, et al., 2022), suggesting that older adults experienced the same or even higher levels of life satisfaction following the pandemic outbreak, despite older adults being at a higher risk of severe illness and mortality from COVID‐19 (Jordan et al., 2020; Shahid et al., 2020). The mechanisms underlying the stability of well‐being across the lifespan during the pandemic are not fully understood (Martire & Isaacowitz, 2021). Researchers should better understand the factors that drive the resilience of older adults in the face of adversity.

Lastly, sex, education, region of residence, residential status, and having a paid job were not statistically significantly associated with trajectories of life satisfaction during the pandemic. These non‐significant results were consistent across samples. However, the results should be interpreted carefully. As our follow‐up analysis suggested (see Tables S5 and S6), most individuals did not experience changes in residential status, support network, and having a paid job from pre‐ to mid‐pandemic periods, and we might not reliably track individuals who faced a loss of resources after the outbreak. Thus, we could overlook people at particularly high risk amid the pandemic and find stability in life satisfaction over time.

## LIMITATIONS

There are several limitations to note. First, the generalizability of the results should be interpreted with caution. Because this study used data from the online survey panels and the data did not include individuals who did not use computers, our findings may not be generalizable to a population with limited resources, such as lower socioeconomic status and older age. Also, the follow‐up rates were not very high, with 42.6% of the 2019 sample and 60.5% of the 2020 sample. Moreover, older adults were more likely to continue to participate in the follow‐up surveys across samples (see Tables S1 and S2). The high dropout ratios and the missing pattern might have violated the missing‐at‐random assumption and could cause biased estimates. However, we found only negligible differences in life satisfaction between the participants who dropped out and those who continued in the 2019 sample. Additionally, we do not know whether the current results were unique to Japan or similar across East Asian or collectivistic countries.

Second, well‐being is considered a multi‐dimensional construct (Diener, 1984; Diener et al., 1999), but this study assessed life satisfaction only. As initial evidence suggested (Aknin, De Neve, et al., 2022), differential patterns of changes might emerge during the pandemic according to components of well‐being.

Third, the baseline survey of the 2020 sample was conducted in February 2020, just after the first case of COVID‐19 was reported in January in Japan. The pandemic might have started to influence people's daily lives through social media during the baseline survey in 2020. Thus, it would have been more accurate to name the time metric in the 2020 sample as the peri‐pandemic period rather than the pre‐pandemic period.

Fourth, the number of observations was limited (i.e., three measurement observations per sample), and the time intervals across surveys were widely spaced, which limited our ability to capture fluctuations and drastic changes in life satisfaction shortly following and during the COVID‐19 pandemic. Life satisfaction might have varied in the short term in response to the coronavirus spread and the government's emergency declarations. In fact, an earlier study adopting monthly assessments detected decreases in well‐being during lockdown and restriction periods (Zacher & Rudolph, 2023). Such longitudinal research with short time intervals would enable us to accurately track changes in well‐being amid the pandemic.

Fifth, the study variables were assessed through self‐reports. To avoid biases, further studies should consider the usage of objective assessments, such as diagnosed diseases and household income. Also, although we considered major risk and protective factors of well‐being, they were not comprehensive enough, and other unobserved factors might account for trajectories of life satisfaction during the pandemic. For example, other personal events could also impact individuals' daily lives.

Finally, we handled missing data due to dropout for continuous resources using maximum likelihood estimation but could not apply this method for categorical resources. Consequently, we chose respondents who continued to participate in the pre‐ and mid‐pandemic surveys in the follow‐up analysis. Alternative statistical models should be reconsidered to estimate changes in both continuous and categorical variables.

## CONCLUSION

To better understand the impact of the COVID‐19 pandemic on individuals' well‐being, this study examined how individuals' life satisfaction changed in Japan before and during the pandemic. Our findings showed that most individuals did not experience a deterioration in life satisfaction following the pandemic outbreak. We further observed that changes in economic and health‐related resources were associated with trajectories of life satisfaction during the pandemic. Some individuals might face a loss of resources for life satisfaction under extremely stressful circumstances. We also found that individuals without a support network before the pandemic displayed steeper increases in life satisfaction after the outbreak. Vulnerable individuals might become resilient and gain new social resources even in times of social‐distancing measures. To better understand resilience and well‐being, researchers should consider how the COVID‐19 pandemic has changed multiple aspects of daily lives, including social, economic, and health‐related, across the globe.

## CONFLICT OF INTEREST STATEMENT

We have no conflicts of interest to disclose.

## ETHICS STATEMENT

We did not apply for ethics approval for the current study because we used publicly available deidentifying datasets.

## FUNDING STATEMENT

This work was supported by the Japan Society for the Promotion of Science [grant number 21H00943], the National Center for Geriatrics and Gerontology [grant numbers 20–58 and 21–17], and the Japan Science and Technology Agency [grant number JPMJSC2106].

## Supporting information


**Figure S1.** Study design and participation in baseline and follow‐up surveys.
**Figure S2.** Daily new confirmed COVID‐19 cases in Japan from 16th January 2019 to 1st March 2022. Source: Ministry of Health, Labour and Welfare. Visualizing the data: Information on COVID‐19 infections. https://covid19.mhlw.go.jp/en/
**Figure S3.** Graphical illustration of hypothetical trajectories of life satisfaction based on linear and piecewise growth models.
**Figure S4.** Average piecewise changes in life satisfaction between the pre‐and mid‐pandemic periods. On average, life satisfaction remained stable before and during the pandemic outbreak. See Table 2 for coefficients estimated in unconditional models without predictors.
**Table S1.** Descriptive characteristics of the continuers and dropouts in the 2019 sample.
**Table S2.** Descriptive characteristics of the continuers and dropouts in the 2020 sample.
**Table S3.** Piecewise growth models for economic satisfaction and self‐rated health.
**Table S4.** Linear and piecewise growth models for life satisfaction: unconditional models without predictors.
**Table S5.** Descriptive characteristics of the continuers.
**Table S6.** Piecewise growth model for life satisfaction: Conditional models with time‐varying categorical predictors.

## Data Availability

The Cabinet Office of Japan initiated the Survey on Satisfaction and Quality of Life and provided the deidentified data. All the datasets are available at https://www5.cao.go.jp/keizai2/wellbeing/manzoku/index.html.
