# Supplementary material for: Stability and change in life satisfaction in Japan before and during the COVID‐19 pandemic
Source: Appl Psychol Health Well Being. 2025 Mar 27;17(2):e70021. doi: 10.1111/aphw.70021 (PMC11949741; doi:10.1111/aphw.70021)
Supplement: Supplementary file 1 — Figure S1. Study design and participation in baseline and follow‐up surveys. Figure S2. Daily new confirmed COVID‐19 cases in Japan from 16th January 2019 to 1st March 2022. Source: Ministry of Health, Labour and Welfare. Visualizing the data: Information on COVID‐19 infections. https://covid19.mhlw.go.jp/en/ Figure S3. Graphical illustration of hypothetical trajectories of life satisfaction based on linear and piecewise growth models. Figure S4. Average piecewise changes in life satisfaction between the pre‐and mid‐pandemic periods. On average, life satisfaction remained stable before and during the pandemic outbreak. See Table 2 for coefficients estimated in unconditional models without predictors. Table S1. Descriptive characteristics of the continuers and dropouts in the 2019 sample. Table S2. Descriptive characteristics of the continuers and dropouts in the 2020 sample. Table S3. Piecewise growth models for economic satisfaction and self‐rated health. Table S4. Linear and piecewise growth models for life satisfaction: unconditional models without predictors. Table S5. Descriptive characteristics of the continuers. Table S6. Piecewise growth model for life satisfaction: Conditional models with time‐varying categorical predictors. [file APHW-17-0-s001.docx]

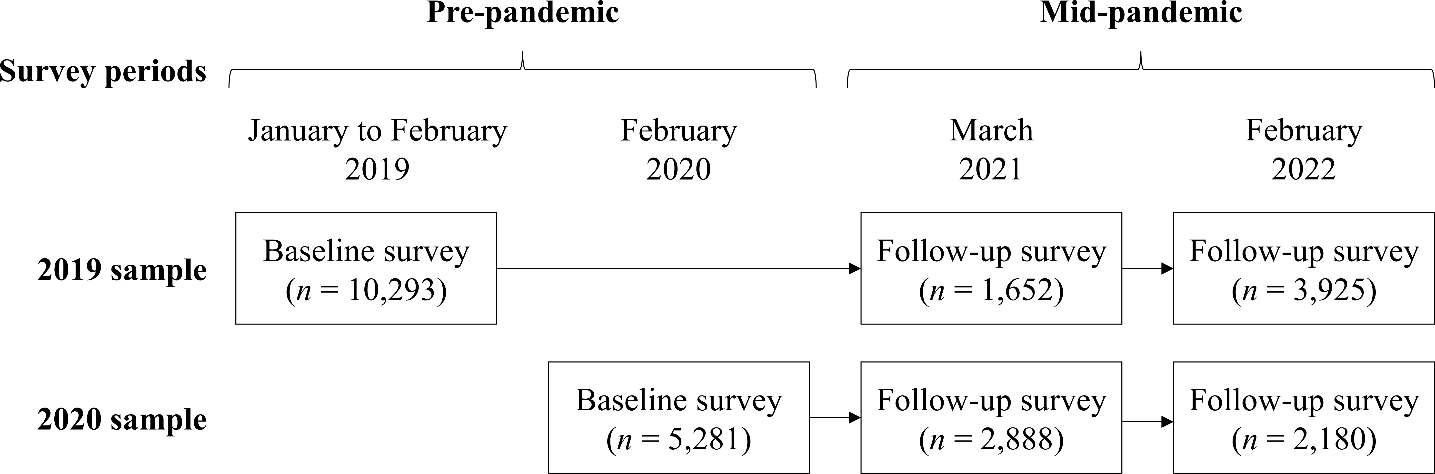


**Supplementary Figure 1.** Study design and participation in baseline and follow-up surveys.

**
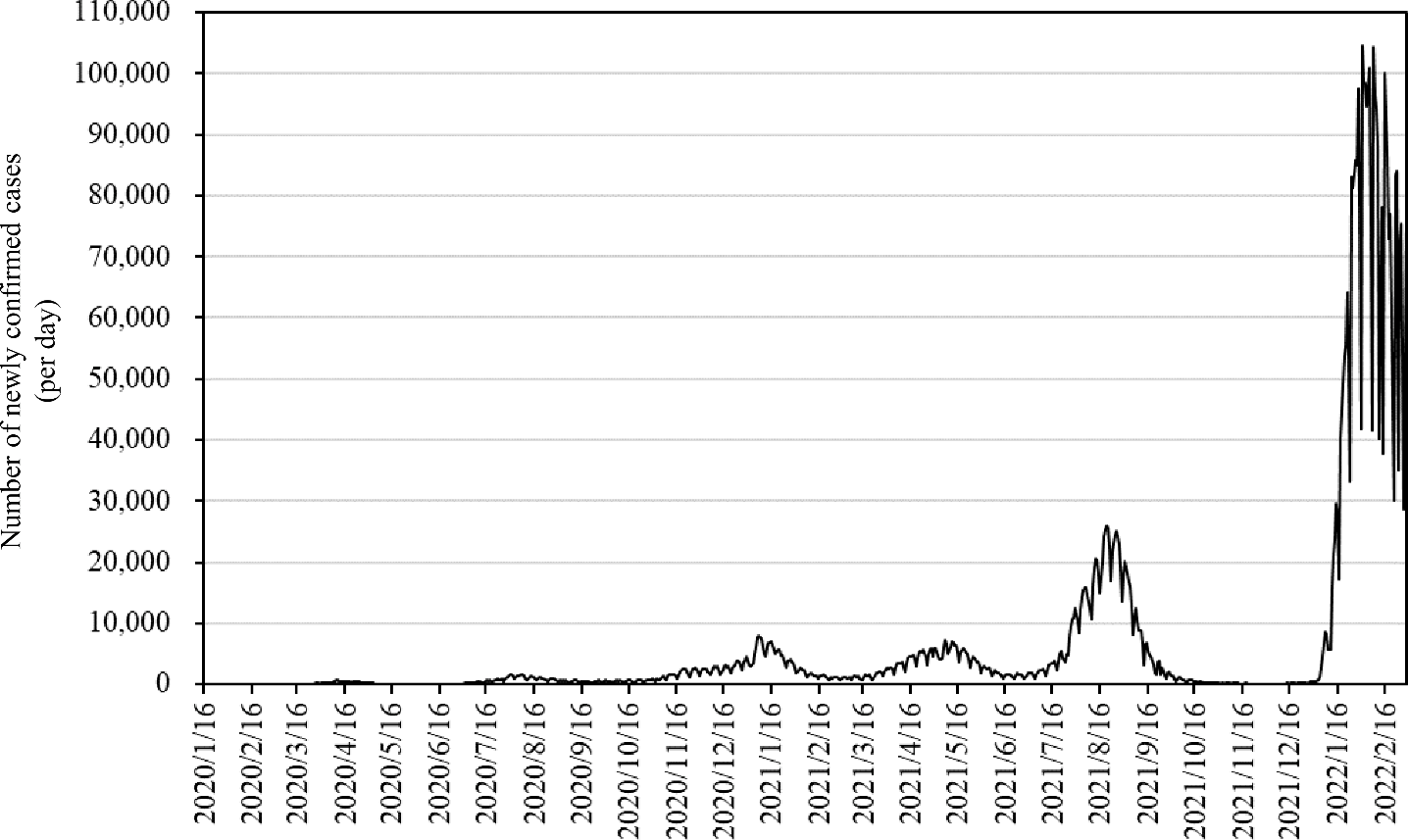
**

**Supplementary Figure 2.** Daily new confirmed COVID-19 cases in Japan from 16^th^ January 2019 to 1^st^ March 2022. Source: Ministry of Health, Labour and Welfare. Visualizing the data: Information on COVID-19 infections. https://covid19.mhlw.go.jp/en/

**
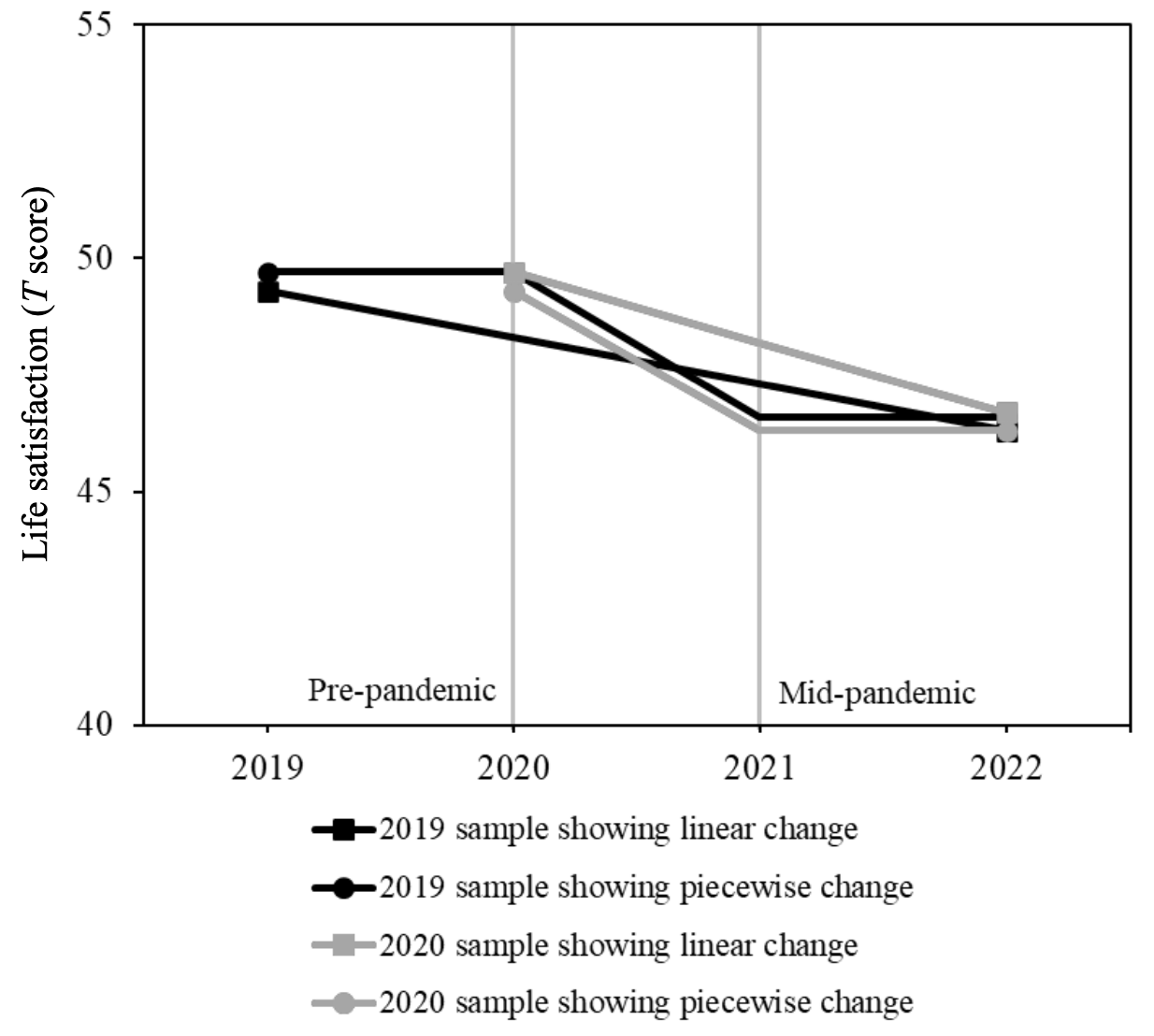
**

**Supplementary Figure 3.** Graphical illustration of hypothetical trajectories of life satisfaction based on linear and piecewise growth models.

**
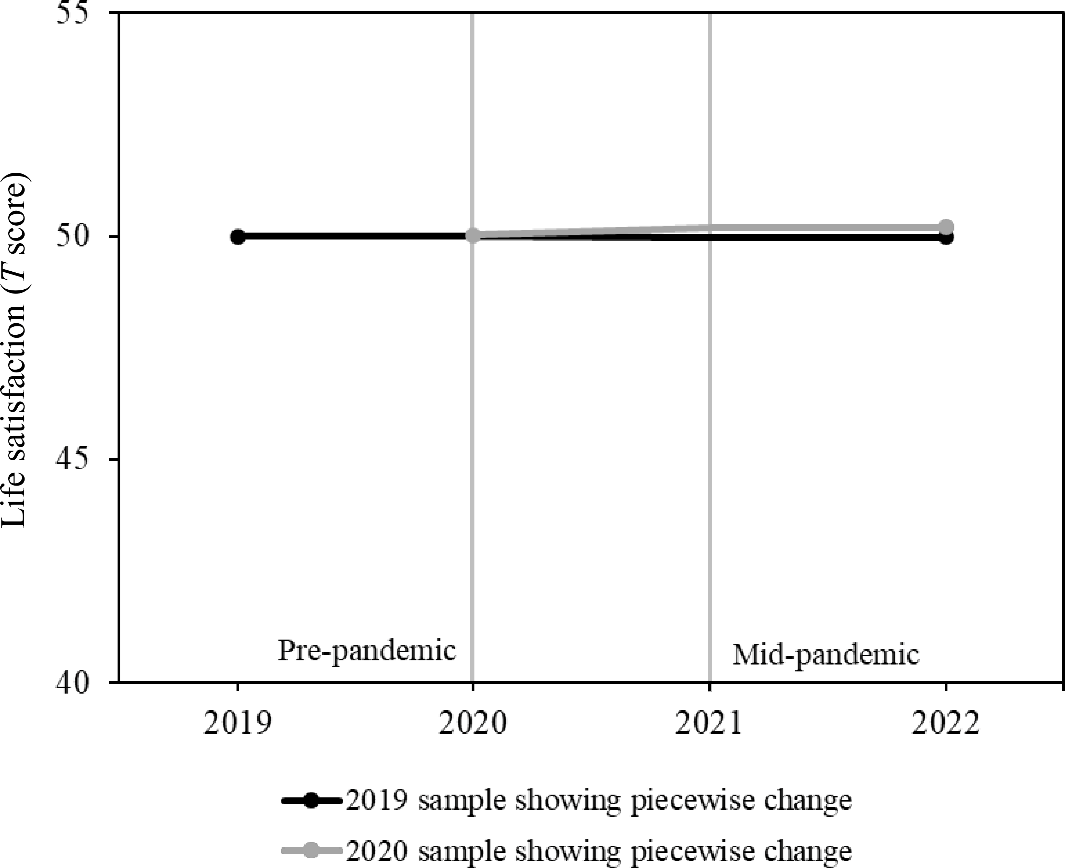
**

**Supplementary Figure 4.** Average piecewise changes in life satisfaction between the pre-and mid-pandemic periods. On average, life satisfaction remained stable before and during the pandemic outbreak. See Table 2 for coefficients estimated in unconditional models without predictors.

**Supplementary Table 1.** Descriptive characteristics of the continuers and dropouts in the 2019 sample.

|  |  | Continuers  (*n* = 4,383) | |  | Dropouts  (*n* = 5,910) | |  | Test of the difference | |
| --- | --- | --- | --- | --- | --- | --- | --- | --- | --- |
| Variables |  | *M* or *n* | *SD* or % |  | *M* or *n* | *SD* or % |  | Statistic | *p* |
| Age | 15 to 24 | 424 | 9.7% |  | 1,368 | 23.1% |  | χ^2^(5) = 493.91 | <.001 |
|  | 25 to 34 | 621 | 14.2% |  | 1,216 | 20.6% |  |  |  |
|  | 35 to 44 | 900 | 20.5% |  | 1,059 | 17.9% |  |  |  |
|  | 45 to 54 | 807 | 18.4% |  | 756 | 12.8% |  |  |  |
|  | 55 to 64 | 920 | 21.0% |  | 835 | 14.1% |  |  |  |
|  | 65+ | 711 | 16.2% |  | 676 | 11.4% |  |  |  |
| Sex | Male | 2,293 | 52.3% |  | 2,809 | 47.5% |  | χ^2^(1) = 23.06 | <.001 |
|  | Female | 2,090 | 47.7% |  | 3,101 | 52.5% |  |  |  |
| Education | Low | 1,451 | 33.1% |  | 1,937 | 32.8% |  | χ^2^(2) = 2.13 | .345 |
|  | Medium | 988 | 22.5% |  | 1,404 | 23.8% |  |  |  |
|  | High | 1,944 | 44.4% |  | 2,569 | 43.5% |  |  |  |
| Region | Metropolitan region | 1,342 | 30.6% |  | 1,945 | 32.9% |  | χ^2^(1) = 6.08 | .014 |
|  | Non-metropolitan region | 3,041 | 69.4% |  | 3,965 | 67.1% |  |  |  |
| Residential status | Living alone | 684 | 15.6% |  | 959 | 16.2% |  | χ^2^(1) = 0.72 | .395 |
|  | Living with others | 3,699 | 84.4% |  | 4,951 | 83.8% |  |  |  |
| Support network | No | 492 | 11.2% |  | 498 | 8.4% |  | χ^2^(1) = 22.68 | <.001 |
|  | Yes | 3,891 | 88.8% |  | 5,412 | 91.6% |  |  |  |
| Paid job | No | 1,184 | 27.0% |  | 1,471 | 24.9% |  | χ^2^(1) = 5.93 | .015 |
|  | Yes | 3,199 | 73.0% |  | 4,439 | 75.1% |  |  |  |
| Economic satisfaction |  | 4.71 | 2.35 |  | 4.64 | 2.33 |  | *t*(10,291) = 1.47 | .141 |
| Self-rated health |  | 2.27 | 1.02 |  | 2.38 | 1.04 |  | *t*(9,549.3) = 5.03 | <.001 |
| Life satisfaction |  | 5.72 | 2.37 |  | 5.83 | 2.31 |  | *t*(9,311.8) = 2.33 | .020 |

*Note*. Descriptive statistics at baseline are presented. Higher values indicate better levels of economic satisfaction, self-rated health, and life satisfaction. Metropolitan region includes three major cities in Japan: Tokyo, Osaka, and Nagoya.

**Supplementary Table 2.** Descriptive characteristics of the continuers and dropouts in the 2020 sample.

|  |  | Continuers  (*n* = 3,193) | |  | Dropouts  (*n* = 2,088) | |  | Test of the difference | |
| --- | --- | --- | --- | --- | --- | --- | --- | --- | --- |
| Variables |  | *M* or *n* | *SD* or % |  | *M* or *n* | *SD* or % |  | Statistic | *p* |
| Age | 15 to 24 | 331 | 10.4% |  | 548 | 26.2% |  | χ^2^(5) = 409.70 | <.001 |
|  | 25 to 34 | 436 | 13.7% |  | 476 | 22.8% |  |  |  |
|  | 35 to 44 | 625 | 19.6% |  | 367 | 17.6% |  |  |  |
|  | 45 to 54 | 524 | 16.4% |  | 254 | 12.2% |  |  |  |
|  | 55 to 64 | 716 | 22.4% |  | 270 | 12.9% |  |  |  |
|  | 65+ | 561 | 17.6% |  | 173 | 8.3% |  |  |  |
| Sex | Male | 1,628 | 51.0% |  | 983 | 47.1% |  | χ^2^(1) = 7.71 | .005 |
|  | Female | 1,565 | 49.0% |  | 1,105 | 52.9% |  |  |  |
| Education | Low | 1,010 | 31.6% |  | 646 | 30.9% |  | χ^2^(2) = 4.70 | .095 |
|  | Medium | 752 | 23.6% |  | 546 | 26.1% |  |  |  |
|  | High | 1,431 | 44.8% |  | 896 | 42.9% |  |  |  |
| Region | Metropolitan region | 1,119 | 35.0% |  | 701 | 33.6% |  | χ^2^(1) = 1.21 | .271 |
|  | Non-metropolitan region | 2,074 | 65.0% |  | 1,387 | 66.4% |  |  |  |
| Residential status | Living alone | 512 | 16.0% |  | 366 | 17.5% |  | χ^2^(1) = 2.03 | .154 |
|  | Living with others | 2,681 | 84.0% |  | 1,722 | 82.5% |  |  |  |
| Support network | No | 329 | 10.3% |  | 159 | 7.6% |  | χ^2^(1) = 10.88 | <.001 |
|  | Yes | 2,864 | 89.7% |  | 1,929 | 92.4% |  |  |  |
| Paid job | No | 755 | 23.6% |  | 371 | 17.8% |  | χ^2^(1) = 26.00 | <.001 |
|  | Yes | 2,438 | 76.4% |  | 1,717 | 82.2% |  |  |  |
| Economic satisfaction |  | 4.81 | 2.31 |  | 4.67 | 2.36 |  | *t*(5,279) = 2.13 | .033 |
| Self-rated health |  | 2.31 | 1.02 |  | 2.38 | 1.06 |  | *t*(5,279) = 2.44 | .015 |
| Life satisfaction |  | 5.83 | 2.29 |  | 5.82 | 2.33 |  | *t*(5,279) = 0.05 | .957 |

*Note*. Descriptive statistics at baseline are presented. Higher values indicate better levels of economic satisfaction, self-rated health, and life satisfaction. Metropolitan region includes three major cities in Japan: Tokyo, Osaka, and Nagoya.

**Supplementary Table 3.** Piecewise growth models for economic satisfaction and self-rated health.

|  | 2019 sample  (*n* = 10,293) | |  | 2020 sample  (*n* = 5,281) | |
| --- | --- | --- | --- | --- | --- |
|  | Economic satisfaction | Self-rated health |  | Economic satisfaction | Self-rated health |
|  | Estimate (*SE*) | |  | Estimate (*SE*) | |
| Fixed effects |  |  |  |  |  |
| Pre-pandemic intercept | 4.67 (0.02)*** | 2.33 (0.02)*** |  | 4.76 (0.03)*** | 2.34 (0.01)*** |
| Mid-pandemic | 0.28 (0.03)*** | —0.11 (0.13)*** |  | 0.18 (0.03)*** | —0.07 (0.01)*** |
| Random effects |  |  |  |  |  |
| Variance of pre-pandemic intercept | 4.05 (0.10)*** | 0.74 (0.02)*** |  | 3.99 (0.12)*** | 0.73 (0.02)*** |
| Covariance of pre-pandemic intercept and mid-pandemic | —0.52 (0.09)*** | —0.10 (0.02)*** |  | —0.44 (0.09)*** | —0.08 (0.02)*** |
| Variance of mid-pandemic | 1.13 (0.13)*** | 0.20 (0.03)*** |  | 0.72 (0.11)*** | 0.10 (0.03)*** |
| Residual | 1.44 (0.06)*** | 0.32 (0.01)*** |  | 1.44 (0.05)*** | 0.35 (0.01)*** |

*Note*. Unstandardized estimates (standard errors in parentheses) are shown. The intercept is centered in early 2020 when the first case of COVID-19 was reported in Japan. Higher values indicate better levels of economic satisfaction and self-rated health.

*** *p* < .001.

**Supplementary Table 4.** Linear and piecewise growth models for life satisfaction: unconditional models without predictors.

|  | 2019 sample  (*n* = 10,293) | |  | 2020 sample  (*n* = 5,281) | |
| --- | --- | --- | --- | --- | --- |
|  | Linear model | Piecewise model |  | Linear model | Piecewise model |
|  | Estimate (*SE*) | |  | Estimate (*SE*) | |
| Fixed effects |  |  |  |  |  |
| Pre-pandemic intercept | 50.00 (0.10)*** | 50.00 (0.10)*** |  | 50.15 (0.13)*** | 50.19 (0.14)*** |
| Time to/from pandemic | 0.01 (0.04) | ― |  | −0.05 (0.08) | ― |
| Mid-pandemic | ― | −0.00 (0.13) |  | ― | −0.16 (0.12) |
| Random effects |  |  |  |  |  |
| Variance of pre-pandemic intercept | 70.51 (1.39)*** | 74.46 (1.73)*** |  | 68.11 (1.99)*** | 70.78 (2.08)*** |
| Covariance of pre-pandemic intercept and time | −0.46 (0.38) | −8.46 (1.58)*** |  | −0.73 (0.89) | −4.64 (1.55)*** |
| Variance of time | 2.14 (0.30)*** | 18.40 (2.30)*** |  | 1.17 (0.66) | 8.48 (1.91)*** |
| Residual | 26.24 (1.04)*** | 25.53 (1.02)*** |  | 28.63 (0.83)*** | 26.54 (0.85)*** |
| Goodness of fit |  |  |  |  |  |
| −2*LL* | 114,636.58 | 114,636.52 |  | 73,040.98 | 73,024.76 |
| *AIC* | 114,648.58 | 114,648.52 |  | 73,052.98 | 73,036.76 |

*Note*. Unstandardized estimates (standard errors in parentheses) are shown. In both 2019 and 2020 samples, life satisfaction score is standardized to a *T* metric (*M* = 50, *SD* =10) based on baseline data of 2019 sample. The intercept is centered in early 2020 when the first case of COVID-19 was reported in Japan; the rate of change over time to/from pandemic is scaled in years. Time in the random effect components indicates time to/from pandemic or mid-pandemic time metric. –2*LL* = –2 log likelihood; *AIC* = Akaike information criterion.

*** *p* < .<.001.

**Supplementary Table 5.** Descriptive characteristics of the continuers.

|  |  | 2019 sample  (*n* = 1,652) | |  | 2020 sample  (*n* = 2,888) | |
| --- | --- | --- | --- | --- | --- | --- |
| Variables |  | *n* | % |  | *n* | % |
| Residential status | Continuously living alone | 201 | 12.2% |  | 418 | 14.5% |
|  | Continuously living with others | 1,324 | 80.1% |  | 2,354 | 81.5% |
|  | Becoming living alone | 76 | 4.6% |  | 72 | 2.5% |
|  | Becoming living with others | 51 | 3.1% |  | 44 | 1.5% |
| Support network | Continuously having no support network | 93 | 5.6% |  | 156 | 5.4% |
|  | Continuously having support network | 1,384 | 83.8% |  | 2,440 | 84.5% |
|  | Losing support network | 89 | 5.4% |  | 159 | 5.5% |
|  | Gaining support network | 86 | 5.2% |  | 133 | 4.6% |
| Paid job | Continuously having no job | 311 | 18.8% |  | 615 | 21.3% |
|  | Continuously having job | 1,154 | 69.9% |  | 1,998 | 69.2% |
|  | Losing job | 101 | 6.1% |  | 185 | 6.4% |
|  | Gaining job | 86 | 5.2% |  | 90 | 3.1% |

*Note.* Study samples participated in online baseline surveys in 2019 or 2020 and were followed up in 2021 and 2022. Continuers indicate individuals who participated in the baseline (i.e., 2019 or 2020) and follow-up (i.e., 2021) surveys. Pre- and mid-pandemic categorical variables were created using observed values in the baseline and 2021 surveys.

**Supplementary Table 6.** Piecewise growth model for life satisfaction: Conditional models with time-varying categorical predictors.

|  |  | 2019 sample  (*n* = 1,652) | |
| --- | --- | --- | --- |
|  |  | Pre-pandemic intercept | Mid-pandemic |
|  |  | Estimate (*SE*) | |
| Fixed effects |  | 46.51 (0.50)*** | 1.34 (0.50)** |
| Age in 2019 | 15 to 24 | 0.17 (0.40) | −1.23 (0.40)** |
|  | 25 to 34 | −0.94 (0.34)** | −0.02 (0.34) |
|  | 35 to 44 | −0.37 (0.34) | −0.02 (0.34) |
|  | 45 to 54 | −0.87 (0.41)* | 1.02 (0.41)* |
|  | 55 to 64 | 0.25 (0.43) | 0.53 (0.43) |
|  | 65+ | 1.75 (0.53)*** | −0.28 (0.53) |
| Sex | Male | −0.58 (0.18)** | 0.32 (0.18) |
|  | Female | 0.58 (0.18)** | −0.32 (0.18) |
| Education | Low | −0.01 (0.10) | −0.02 (0.24) |
|  | Medium | −0.15 (0.26) | 0.30 (0.26) |
|  | High | 0.17 (0.24) | −0.20 (0.24) |
| Region in 2019 | Metropolitan region | 0.02 (0.19) | −0.08 (0.19) |
|  | Non-metropolitan region | −0.02 (0.19) | 0.08 (0.19) |
| Residential status | Continuously living alone | 0.54 (0.48) | −0.93 (0.48) |
|  | Continuously living with others | 1.01 (0.37)** | −0.69 (0.38) |
|  | Becoming living alone | −0.89 (0.64) | 0.65 (0.65) |
|  | Becoming living with others | −0.66 (0.75) | 0.98 (0.77) |
| Support network | Continuously having no support network | −1.41 (0.60)* | −0.31 (0.60) |
|  | Continuously having support network | 1.95 (0.36)*** | −0.39 (0.36) |
|  | Losing support network | −0.23 (0.60) | −0.27 (0.60) |
|  | Gaining support network | −0.31 (0.61) | 0.97 (0.61) |
| Paid job | Continuously having no job | 0.38 (0.41) | −0.26 (0.41) |
|  | Continuously having job | 0.73 (0.33)* | −0.50 (0.33) |
|  | Losing job | −0.05 (0.55) | 0.44 (0.55) |
|  | Gaining job | −1.06 (0.59) | 0.33 (0.59) |
| Economic satisfaction | Pre-pandemic | 3.34 (0.11)*** | 0.05 (0.11) |
|  | Mid-pandemic | −0.71 (0.17)*** | 3.66 (0.17)*** |
| Self-rated health | Pre-pandemic | 2.20 (0.26)*** | −0.19 (0.26) |
|  | Mid-pandemic | −0.32 (0.17) | 0.74 (0.17)*** |
| Random effects (variance-covariance matrix) | |  | |
|  | Pre-pandemic intercept | 19.43 (1.89)*** |  |
|  | Mid-pandemic | −4.72 (1.70)** | 4.45 (2.27)* |
|  | Residual variance | 25.84 (1.04)*** |  |

*Note*. Unstandardized estimates (standard errors in parentheses) are shown. As the model using data from the 2020 sample did not converge, the results are not reported. Pre- and mid-pandemic continuous predictors were estimated using piecewise growth models. Pre- and mid-pandemic categorical predictors were created using observed values in the baseline and 2021 surveys. Life satisfaction score is standardized to a *T* metric (*M* = 50, *SD* =10) based on baseline data of 2019 sample (i.e., *M* = 5.78, *SD* = 2.34). The intercept is centered in early 2020 when the first case of COVID-19 was reported in Japan. Categorical predictors were effect-coded so that each coefficient represents a deviation from the grand mean. Continuous predictors were centered at the sample means. Mid-pandemic economic satisfaction and self-rated health are standardized. Positive values indicate better levels of life satisfaction than the average levels.

* *p* < .05, ** *p* < .01, *** *p* < .001.
